# Clinical efficacy of OS‐01 peptide formulation in reducing the signs of periorbital skin aging

**DOI:** 10.1111/ics.13042

**Published:** 2025-01-09

**Authors:** Alessandra Zonari, Lear E. Brace, Fanghua Li, Nathaniel H. O. Harder, Claire Harker, Carolyn Jacob, Joely Kaufman, Suneel Chilukuri, Carolina R. Oliveira, Mariana Boroni, Juliana L. Carvalho

**Affiliations:** ^1^ OneSkin, Inc. San Francisco California USA; ^2^ Chicago Cosmetic and Dermatologic Research PLLC Chicago Illinois USA; ^3^ Skin Research Institute Miami Florida USA; ^4^ Refresh Dermatology Houston Texas USA; ^5^ Bioinformatics and Computational Biology Lab Brazilian National Cancer Institute (INCA) Rio de Janeiro Rio de Janeiro Brazil; ^6^ Genomic Sciences and Biotechnology Program Catholic University of Brasilia Brasília Federal District Brazil; ^7^ Laboratory of Interdisciplinary Biosciences, Faculty of Medicine University of Brasília Brasília Federal District Brazil

**Keywords:** cellular senescence, clinical study, peptides, skin health, skincare, wrinkles

## Abstract

**Background:**

The aging of the skin, particularly around the periorbital region, is a complex process characterized by the accumulation of senescent cells, decreased collagen production, and reduced skin elasticity, leading to visible signs such as fine lines, wrinkles, and sagging.

**Objective:**

This study investigates the efficacy of a novel topical formulation, OS‐01 EYE, containing the senomorphic peptide, OS‐01, along with other active ingredients, in improving the skin around the eyes.

**Methods:**

A 12‐week clinical study was conducted with 22 participants who applied OS‐01 EYE twice daily. Assessments included bioinstrumental measurements of skin hydration, barrier function, firmness, and elasticity, as well as expert photographs grading fine lines, wrinkles, under‐eye puffiness, and dark circles.

**Results:**

After 12 weeks of product use, transepidermal water loss (TEWL) decreased by 17.33%, hydration increased by 32.49%, skin firmness improved by 10.19%, and elasticity increased by 25.58%, compared to baseline, which was all statistically significant. Expert grading revealed a decrease in fine lines and wrinkles, under‐eye puffiness, and dark circles over the study period. Furthermore, subjective assessments showed that 95.46% of participants reported improvements in overall appearance.

**Conclusion:**

The OS‐01 EYE formulation was found to be safe and effective in mitigating the visible signs of aging in the periorbital region, making it a potent treatment option for enhancing periorbital skin health, function, and appearance.

## Graphical abstract

In a 12‐week clinical study, a formulation containing the senotherapeutic peptide OS‐01 effectively reduced fine lines and enhanced skin hydration, firmness, and elasticity in the periorbital region. Additionally, expert evaluations and self‐reported participant feedback validated the formulation's efficacy and safety.

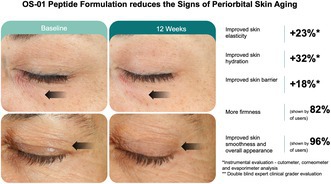

## Résumé

**Contexte:**

Le vieillissement de la peau, en particulier dans la région périorbitaire, est un processus complexe caractérisé par l'accumulation de cellules sénescentes, une diminution de la production de collagène et une réduction de l'élasticité de la peau, ce qui entraîne des signes visibles tels que les ridules, les rides et l'affaissement.

**Objectif:**

Cette étude examine l'efficacité d'une nouvelle formulation topique, OS‐01 EYE, contenant le peptide sénomorphique OS‐01, ainsi que d'autres principes actifs, dans l'amélioration de la peau autour des yeux.

**Méthodes:**

Une étude clinique de 12 semaines a été menée auprès de 22 participants qui ont appliqué l'OS‐01 EYE deux fois par jour. Les évaluations comprenaient des mesures bioinstrumentales de l'hydratation de la peau, de la fonction de barrière, de la fermeté et de l'élasticité, ainsi que des photographies d'experts évaluant les ridules, les rides, les poches sous les yeux et les cernes.

**Résultats:**

Après 12 semaines d'utilisation du produit, la perte d'eau transépidermique (Transepidermal Water Loss, TEWL) a diminué de 17,33 %, l'hydratation a augmenté de 32,49 %, la fermeté de la peau s'est améliorée de 10,19 % et l'élasticité a augmenté de 25,58 %, par rapport à l'entrée dans l'étude, des éléments tous statistiquement significatifs. L'évaluation des experts a révélé une diminution des ridules et des rides, des poches sous les yeux et des cernes au cours de la période de l'étude. En outre, les évaluations subjectives ont montré que 95,46 % des participants ont rapporté une amélioration de l'apparence générale.

**Conclusion:**

La formulation OS‐01 EYE s'est avérée sûre et efficace pour atténuer les signes visibles du vieillissement dans la région périorbitaire, ce qui en fait une alternative de traitement puissante pour améliorer la santé, la fonction et l'apparence de la peau périorbitaire.

## INTRODUCTION

Skin aging is a multifaceted process influenced by internal and external stimuli that affect the skin at the cellular and molecular level [1]. These changes lead to visible alterations, such as fine lines, wrinkles, sagging, and dry skin [2]. Intrinsic factors contributing to skin aging include the accumulation of senescent cells [2, 3, 4], melanogenesis alteration [4], reduced collagen production [5], and reduced proliferation of basal keratinocytes [6]. Additionally, the secretion of senescence‐associated secretory phenotype (SASP) factors by senescent fibroblasts contributes to the degradation of the extracellular matrix by activating matrix metalloproteinases, resulting in a loss of skin firmness and resilience, which manifests as wrinkling and sagging [3, 5]. These senescent‐mediated phenotypes highlight cellular senescence as an actionable molecular target to improve skin health and function.

Recent studies have shown that the periorbital region often ages at a faster rate than other regions of the skin and thus exhibits earlier signs of aging [7, 8]. The skin around the eyes has been found to have a thinner epidermis and stratum corneum compared to the cheek and forehead, as demonstrated by histological analysis [7, 9]. Gene expression pathway studies, gene age prediction modelling, and visual observations further confirm that the aging process in the periorbital region differs from other facial sites [7]. Due to these unique properties, the skin around the eyes requires specialized treatments to target and reduce signs of aging and functional decline.

Senescent cells accumulate in the epidermal and dermal layers of the skin over time. Multiple studies have demonstrated that these cells are not merely a byproduct of aging but actively contribute to skin aging, induce age‐related changes in neighbouring cells, and cause tissue dysfunction through SASP [10]. Reducing the presence of senescent cells can diminish signs of aging and improve overall skin health. As shown by Chung et al., the topical application of the senomorphic molecule rapamycin showed promising results as an ‘anti‐aging’ treatment, reducing biomarkers of aging and improving skin function [11]. In agreement with this study, previous research from our group demonstrated the benefits of a senomorphic peptide, OS‐01 (Peptide 14), in promoting a healthy skin phenotype: OS‐01 reduces skin DNA methylation age by preventing cellular senescence progression, enhances DNA repair, and reduces SASP‐associated gene expression in both 3D skin in vivo models and ex vivo skin [12]. In a double‐blinded, vehicle‐controlled clinical study, our group confirmed the safety and efficacy of OS‐01 in treating aging‐related skin changes when applied topically [13].

With these findings, we developed a novel formulation, OS‐01 EYE, which combines a higher concentration of OS‐01 with other active ingredients to specifically target aging in the periorbital region. This formula includes actives known to boost collagen production and increase moisturization, such as acetyl hexapeptide‐8 [14], *Fucus vesiculosus* extract [15, 16], *Moringa oleifera s*eed extract [17], and hyaluronic acid. The combination of actives with OS‐01 peptide aims to create a synergistic formulation strategically designed to mitigate the exacerbated effects of aging in the delicate periorbital region. In this manuscript, we present a 12‐week clinical study evaluating the safety and effectiveness of OS‐01 EYE in improving skin condition.

## METHODS

### Clinical study design overview

A 12‐week clinical trial was designed to evaluate the effectiveness of a topical formulation containing the OS‐01 peptide (OS‐01 EYE from OneSkin Inc.) in improving skin conditions around the eye. Before initiation, the study was approved by the Veritas Institutional Review Board (2022‐3082‐11740‐4), and informed consent was obtained from each volunteer prior to initiating the study. This clinical study was conducted in accordance with the International Conference of Harmonization Tripartite Guideline on Good Clinical Practice, applicable FDA regulations/guidelines set forth in 21 CFR Parts 11, and 50, and standard practices of ALS Beauty and Personal Care International Research Services, Inc.

Twenty‐two participants (Table 1) who met the inclusion criteria (detailed in Table S1) were instructed to use a dime‐sized amount of OS‐01 EYE (OneSkin Inc.) twice daily for 12 weeks. A cleanser (PREP from OneSkin Inc.), a face moisturizer (OS‐01 FACE from OneSkin Inc.), and sunscreen (Neutrogena Oil‐free moisture SPF 35 from Neutrogena Corp.) were also provided for participant use throughout the study period to standardize daily skincare routine and reduce variability. No adverse events were reported during the study period.

**TABLE 1:** Subject demographics.

No. of participants	Age	Race	Gender	Fitzpatrick skin type
16	47–65	Caucasian	Female	I–III
6	47–64	Caucasian	Male	II–III

Product efficacy was determined by analysis of results from instrumental assessments, group‐blinded expert clinical grading image analysis executed at baseline, 4, and 12 weeks of treatment. Subjective questionnaires were also used to evaluate user perception of product performance.

### Study methods

**FIGURE 1:**
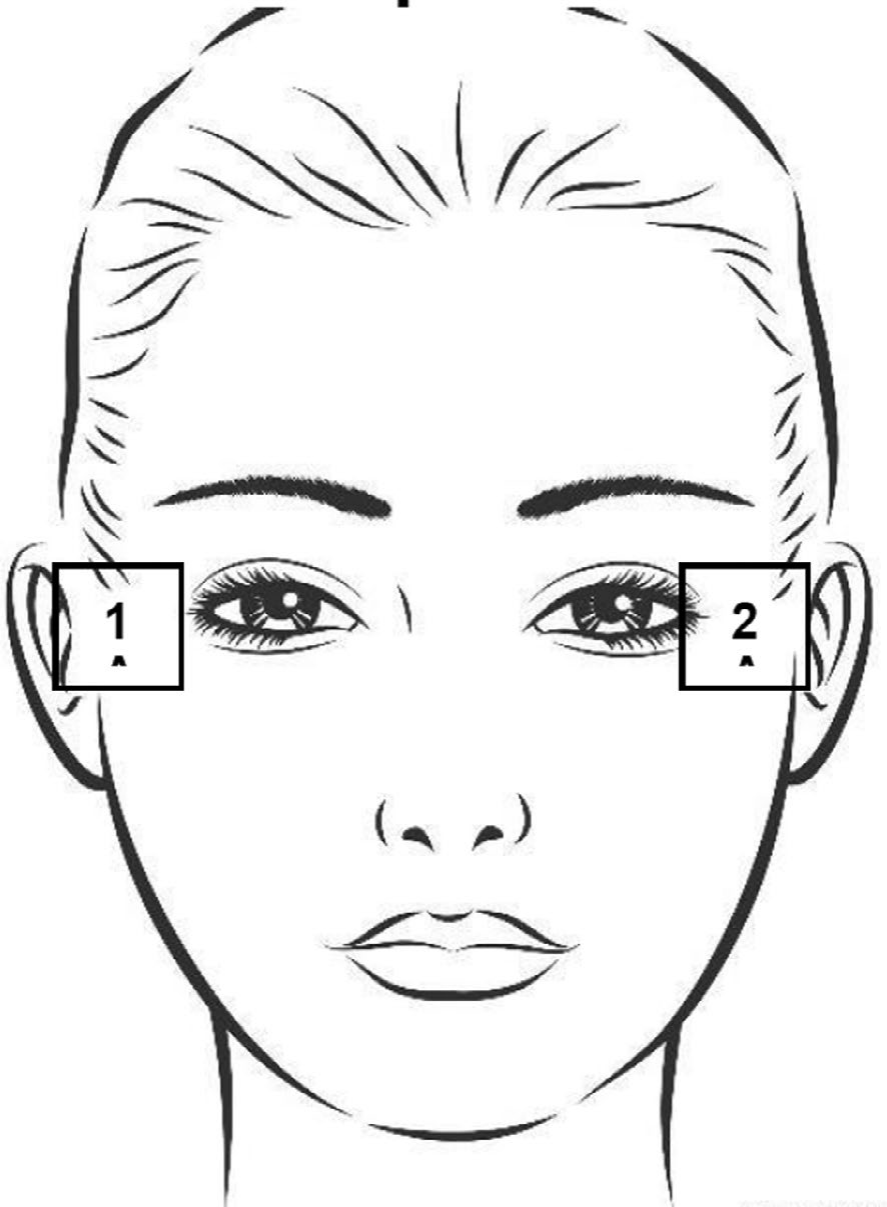
Schematic representation of test sites.

Subjects underwent assessments at baseline (after a 7‐day washout period), 4, and 12 weeks after product usage. The assessments were performed on the outside corner of each eye (Figure 1). At the start of each visit, subjects cleansed their face with the cleanser described above and then equilibrated for 15 min before any photos or measurements were conducted. All measurements were done at room temperature (20–24°C).

#### Bioinstrumental method to measure skin hydration

Changes in skin conductance, impedance, or capacitance are used to study epidermal hydration in vivo [18]. When skin is hydrated, conductance and capacitance increase, and impedance decreases. When measuring capacitance, the measuring capacitor shows changes in capacitance according to the moisture content of the tissue. Corneometer CM 825 (Courage and Khazaka, Germany) was used to measure the electrical capacitance/hydration or conductance/hydration, respectively, of the skin. Three replicate measurements were taken on the outside corner of each eye at each time interval.

#### Bioinstrumental method to measure skin barrier function

Trans‐epidermal water loss (TEWL) is a measure of skin barrier function [19]. The evaporimeter probe has two sensors, which measure the vapour pressure gradient arising within the chamber and between the skin and the surrounding air. TEWL was measured using the DermaLab Evaporimeter (Cortex Technology, Hadsund, Denmark). Decreases in TEWL post‐barrier disruption indicate improvement in skin barrier function, as less water is lost through the skin barrier. TEWL measurements were taken twice from the designated sites at each time interval.

#### Bioinstrumental method to measure firmness and elasticity of periorbital skin

The biomechanical properties of human skin are a complex combination of elastic (elastin fibers) and viscous (collagen fibers and surrounding intercellular ground substance) components [20]. The Cutometer allows the measurement of the viscoelastic properties of the skin *in vivo*. The Cutometer MPA 580 (Courage and Khazaka, Germany) was used to measure skin firmness and elasticity.

#### Clinical photography for before and after images and analyses

Clinical photographs of subjects' faces (frontal, left lateral, and right lateral) were taken with the Canfield VISIA‐CR system using the Standard 1 modality.

#### Live expert grading

Each subject had a test site evaluated by an expert grader, blinded to the previous scores, following parameters using the assigned grading scale, half‐point increments were used (Sup. Table 3).

#### Self‐assessment questionnaire

Each subject was instructed to complete a self‐assessment questionnaire provided by the Sponsor at baseline, week 4, and week 12 treatment intervals. The question asked was: In your opinion, in what manner has the tested product improved the following skin parameters? The parameters were: general appearance, skin radiance, skin firmness/elasticity, skin hydration, skin roughness (tone and texture), dark spots (lightening of hyperpigmentation), and puffiness. Participants' overall satisfaction was evaluated using a 5‐point scale, with scores of 1 (insufficient), 2 (poor), 3 (sufficient), 4 (good), and 5 (excellent).

### Statistical analyses

Statistical analyses tested the hypothesis that the pre‐treatment values of each parameter are significantly improved compared to post‐treatment values. GraphPad Prism (Version 10.3.1) was used to generate the graphs and perform statistical analysis. Normality was confirmed using the Shapiro–Wilk test. One‐way‐ANOVA with Tukey's multiple comparisons test was used to determine the significant difference between each time‐point. The percentage of subjects responding in favour of the test is reported in the tables. Statistical significance for all tests was declared if *p*‐value ≤0.05.

#### Post‐hoc power analysis

To enhance the generalizability of the analysis, we performed a post‐hoc power analysis on all instrumental and expert grading outcomes (i.e., skin hydration, firmness, elasticity, and TEWL (barrier function), fine lines and wrinkles, puffiness, dark circles), using PASS 2024 (NCSS, LLC. Kaysville, Utah, USA). We applied a two‐sided, paired‐difference *t*‐test, with a Type I error rate (*α*) of 0.05, using the mean and standard deviation of the paired difference distribution (baseline vs. week 12) as input parameters [21]. Additionally, we calculated Cohen's *d* for each of the 7 outcomes to further support the interpretability of our findings, providing insight into the effect sizes observed across the variables.

## RESULTS

Under the study conditions, 22 healthy participants (Table 1), ages 47–65, completed the baseline, week 4, and week 12 assessments of the 12‐week clinical study. No adverse events were reported during the study period, and no participants dropped out.

### OS‐01 EYE formulation strengthens the skin barrier and improves hydration, firmness, and elasticity measured by instrumental analysis

**FIGURE 2:**
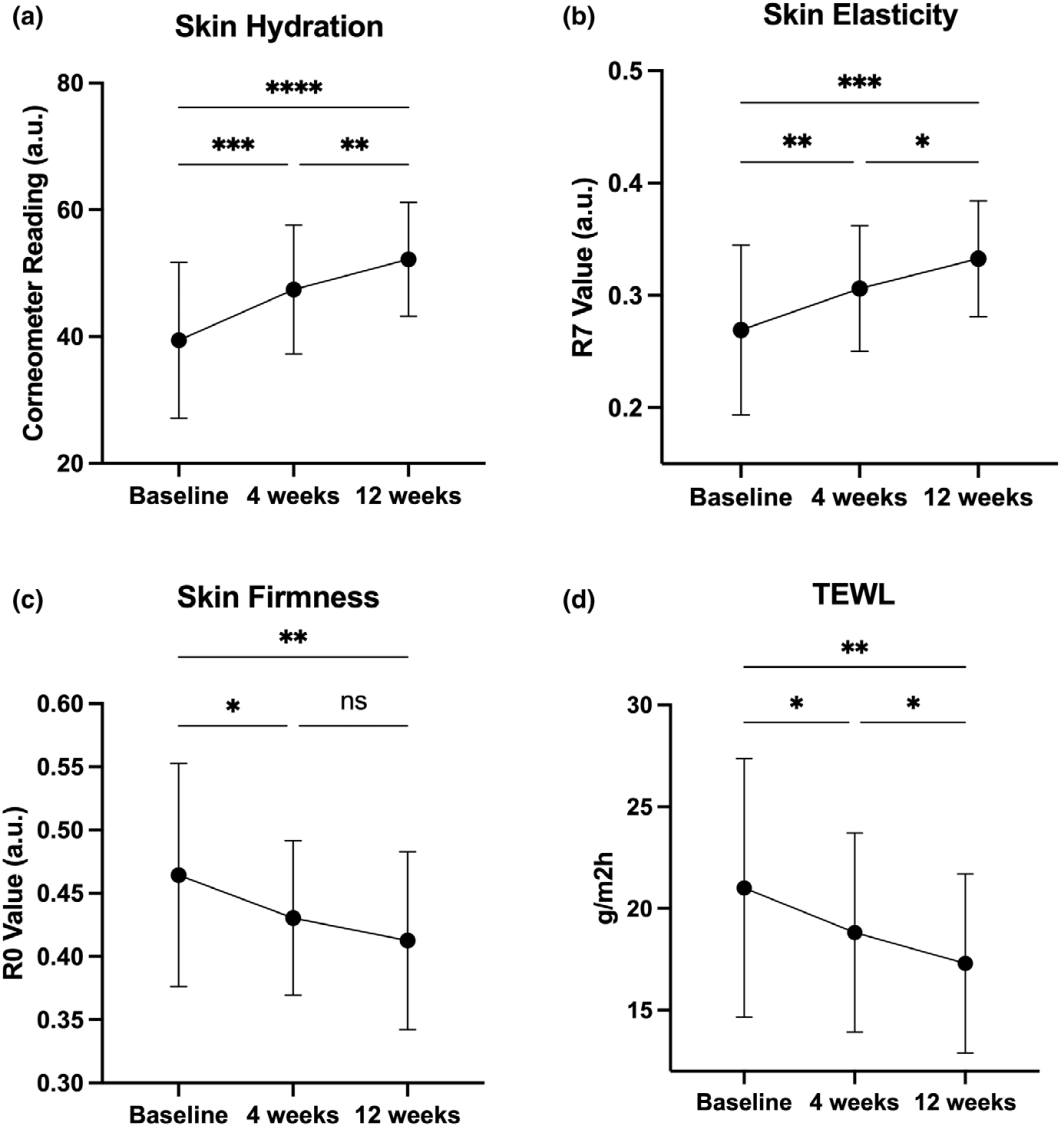
Effects of the OS‐01 EYE formulation using different instrument measurements. (a) Skin hydration measured by Corneometer, (b) skin Elasticity measured by Cutometer (R7 values), (c) Skin Firmness measured by Cutometer (R0 values), and (d) TEWL measured by Evaporimeter. Measurements were taken at baseline, 4 and, 12 weeks. All data are shown as mean ± SD, * indicates statistical significance, *p < 0.05, **p < 0.01, ***p < 0.001, ****p < 0.0001.

At week 4 and week 12, skin barrier function, hydration, firmness, and elasticity were evaluated using evaporimeter, corneometer, and cutometer analysis (Table 2 and Figure 2). Notably, all parameters at both time points showed progressive, statistically significant improvements compared to the baseline measurements. The mean percent improvement for hydration rose to 20.37% (*p* < 0.001) at week 4 and continued to improve, reaching a 32.49% (*p* < 0.001) mean percent improvement at week 12 observed in 87.50% of the participants. Both skin elasticity and firmness showed significant improvements as early as 4 weeks of product use (13.75% and 6.29%, respectively) with further improvements observed by week 12, 25.58% for elasticity and 10.19% for skin firmness observed in 86.36% and 72.73% of the participants. Transepidermal water loss (TEWL), an indicator of skin barrier function, also showed significant improvement at both week 4 (10.07%) and week 12 (17.33%) compared to baseline, observed in 69.57% of the participants after 12 weeks.

**TABLE 2:** Instrumental analysis.

Assessment	Time point	*n*	OS‐01 EYE formulation				
			Mean ± SD	Mean percent improvement *from baseline mean*	Percent of subjects showing improvement *from baseline*	*p*‐Value, *time‐point* versus *baseline*	*p*‐Value, *4 W* versus *12 W*
Hydration (Corneometer)	Baseline	22	30.41 ± 12.30 a.u.				
	Week 4	22	47.43 ± 10.14 a.u.	20.37%	83.33%	<0.001*	
	Week 12	22	52.21 ± 8.97 a.u.	32.49%	87.50%	<0.001*	<0.0001*
Skin Elasticity R7 (Cutometer)	Baseline	22	0.2692 ± 0.0755				
	Week 4	22	0.3062 ± 0.0558 a.u.	13.75%	77.27%	0.007*	
	Week 12	22	0.3326 ± 0.0514 a.u.	25.58%	86.36%	<0.001*	0.049*
Skin Firmness R0 (Cutometer)	Baseline	22	0.4593 ± 0.0914 a.u.				
	Week 4	22	0.4304 ± 0.0611 a.u.	6.29%	68.18%	0.039*	
	Week 12	22	0.4125 ± 0.0704 a.u.	10.19%	72.73%	0.007*	NS
TEWL (Evaporimeter)	Baseline	22	20.92 ± 6.43 g/m^2^ h				
	Week 4	22	18.81 ± 4.90 g/m^2^ h	10.07%	60.87%	0.040*	
	Week 12	22	17.30 ± 4.40 g/m^2^ h	17.33%	69.57%	0.007*	0.046*

Abbreviation: NS, not significant.

* Statistically significant *p* < 0.05.

### OS‐01 EYE improves the appearance of fine lines and wrinkles according to expert grading

**FIGURE 3:**
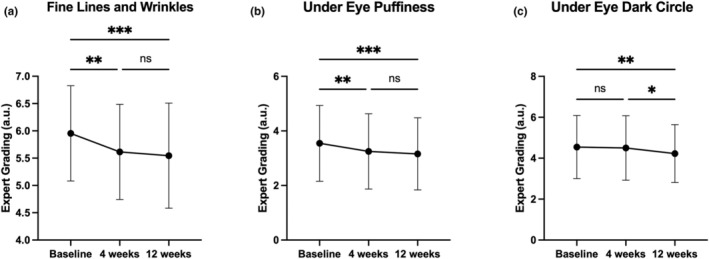
Graphic representation of the expert grading analysis on Visia‐CR photographs. (a) Fine Line and Wrinkles, (b) Under Eye Puffiness, and (c) Under Eye Dark Circle. Measurements were taken at baseline, 4 and 12 weeks. All data are shown as mean ± SD, * indicates statistical significance, *p < 0.05, **p < 0.01, and ***p < 0.001.

At week 4 and week 12, clinical photographs of each study participant's frontal, right lateral, and left lateral (Figure S1) faces were taken using the Canfield VISIA‐CR system for expert grading (Table 3 and Figure 3). At week 4, 45.45% of the study subjects showed improvements in the appearance of fine lines and wrinkles, and 50.00% showed decreases in under‐eye puffiness (*p* = 0.002 and *p* < 0.001, respectively). By week 12, a higher percentage of study subjects (59.09%) showed significant improvements with a mean percent improvement of 6.87% for the appearance of fine lines and wrinkles, and a mean percent improvement of 10.90% for under‐eye puffiness (*p* < 0.001). No significant improvement was observed in reducing under‐eye dark circles at week 4 post‐treatment interval; however, a statistically significant improvement (*p* < 0.001) was observed at week 12 in 50.00% of the participants.

**TABLE 3:** Expert grading of VISIA‐CR photographs.

Assessment	Time point	*n*	OS‐01 EYE formulation				
			Mean ± SD	Mean percent improvement *from BL mean*	Percent of subjects showing improvement *from BL*	*p*‐Value, *TX* versus *BL*	*p*‐Value, *4 W* versus *12 W*
Fine lines and wrinkles	Baseline	22	5.95 ± 0.87 a.u.				
	Week 4	22	5.61 ± 0.87 a.u.	5.73%	45.45%	0.002*	
	Week 12	22	5.55 ± 0.96 a.u.	6.87%	59.09%	<0.001*	NS
Under‐eye puffiness	Baseline	22	3.55 ± 1.39 a.u.				
	Week 4	22	3.25 ± 1.38 a.u.	8.33%	50.00%	0.001*	
	Week 12	22	3.16 ± 1.32 a.u.	10.90%	59.09%	<0.001*	NS
Under‐eye dark circles	Baseline	22	4.55 ± 1.54 a.u.				
	Week 4	22	5.50 ± 1.57 a.u.	1.00%	9.09%	NS	
	Week 12	22	4.23 ± 1.41 a.u.	7.00%	50.00%	0.001*	0.011*

Abbreviation: NS, not significant.

* Statistically significant *p* < 0.05.

**FIGURE 4:**
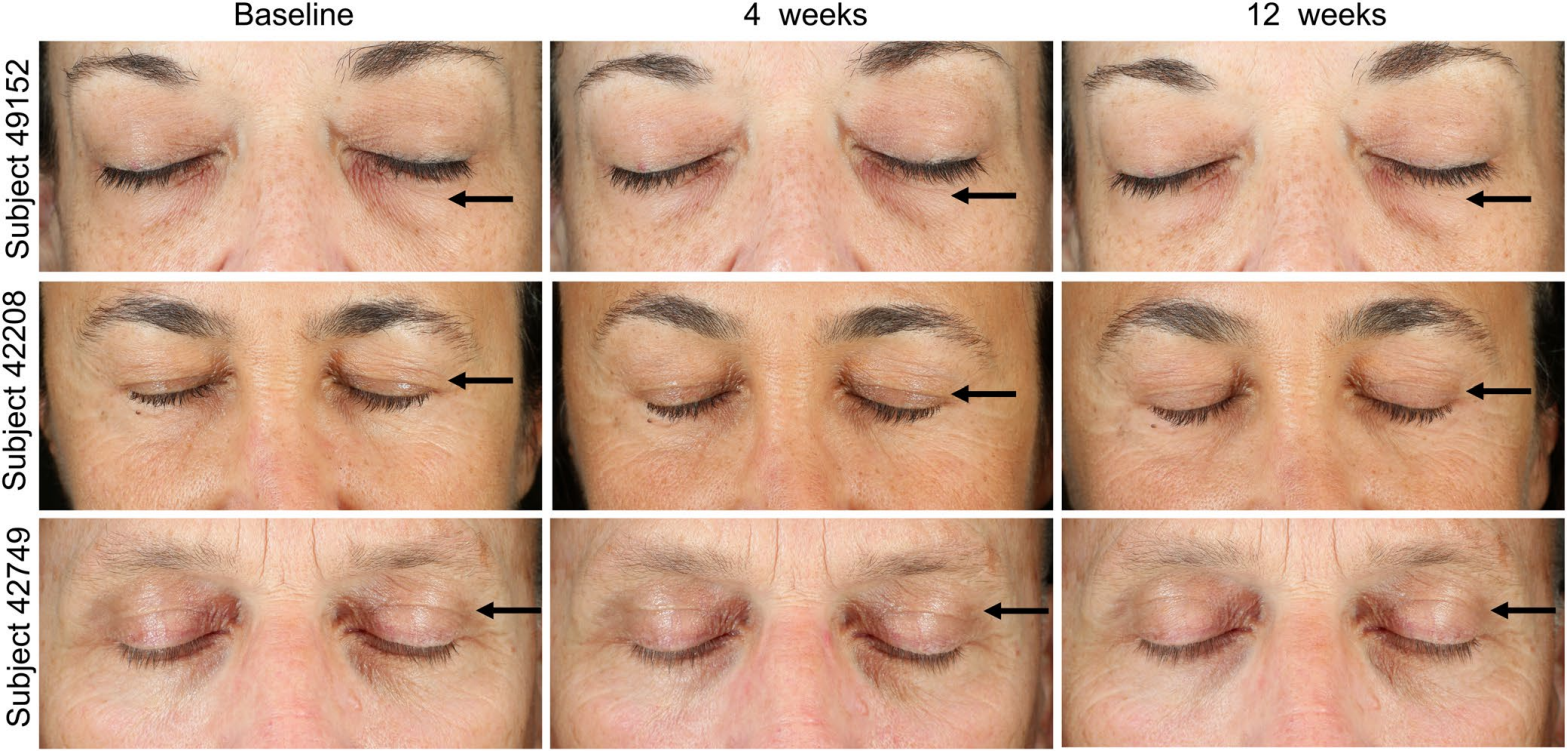
Representative images of the front‐view of the eyes showing the skin‐surface changes of the subjects. [Colour figure can be viewed at wileyonlinelibrary.com]

**FIGURE 5:**
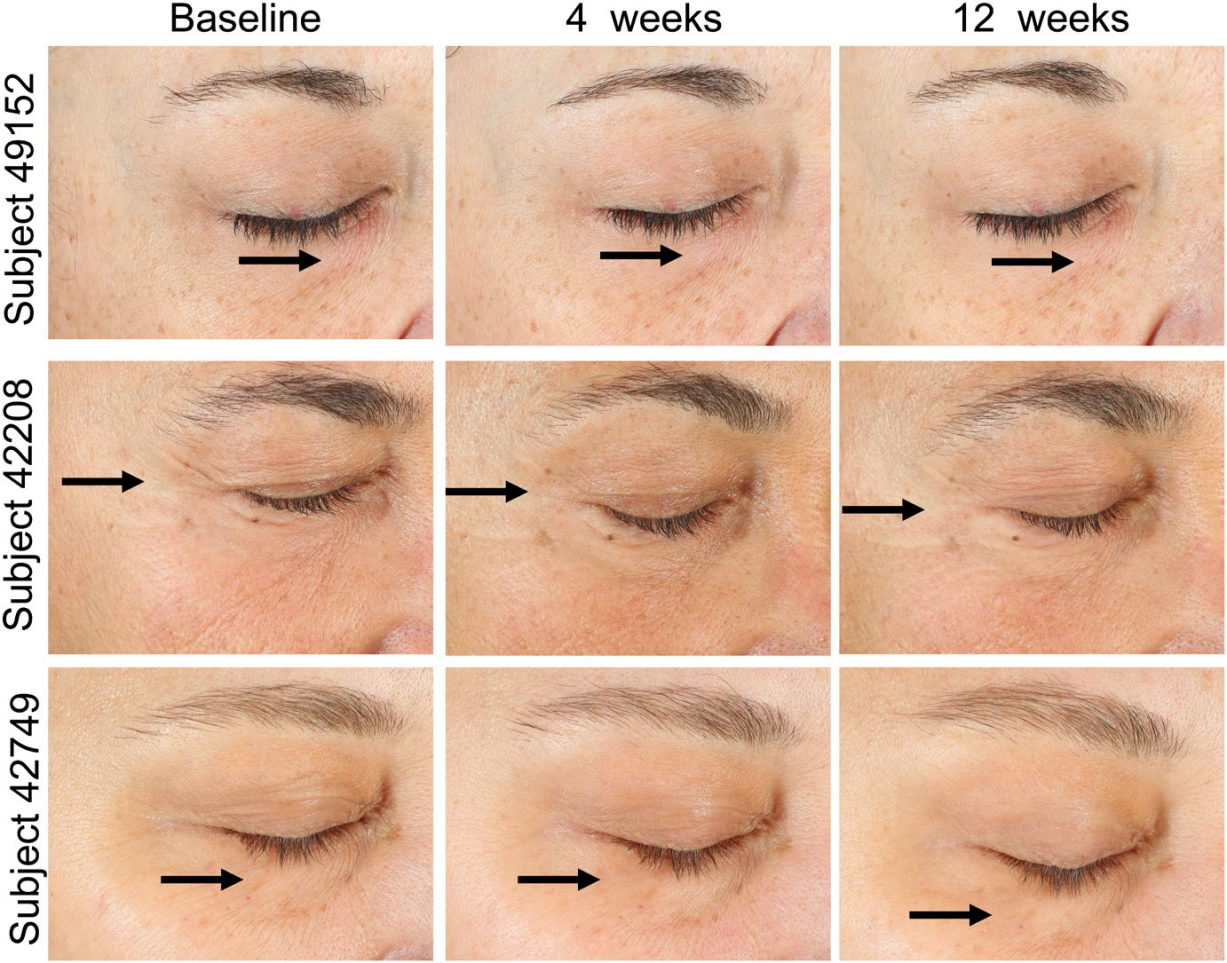
Representative images of the right side of the eyes showing the skin‐surface changes of the subjects. [Colour figure can be viewed at wileyonlinelibrary.com]

The findings reported by blinded expert graders are visible in representative images of subjects and demonstrate visual confirmation of the improvements promoted by OS‐01 EYE. Frontal pictures of three subjects displayed reduced appearances of fine lines and wrinkles, as indicated by the arrows (Figure 4). These improvements were maintained and intensified through week 12. Such improvements were also visible in the representative right (Figure 5) and left lateral (Figure S1) images, where arrows indicate regions of interest with more even skin texture and reduced wrinkles.

### Subjects notice an improvement in different skin parameters after 4 and 12 weeks

**FIGURE 6:**
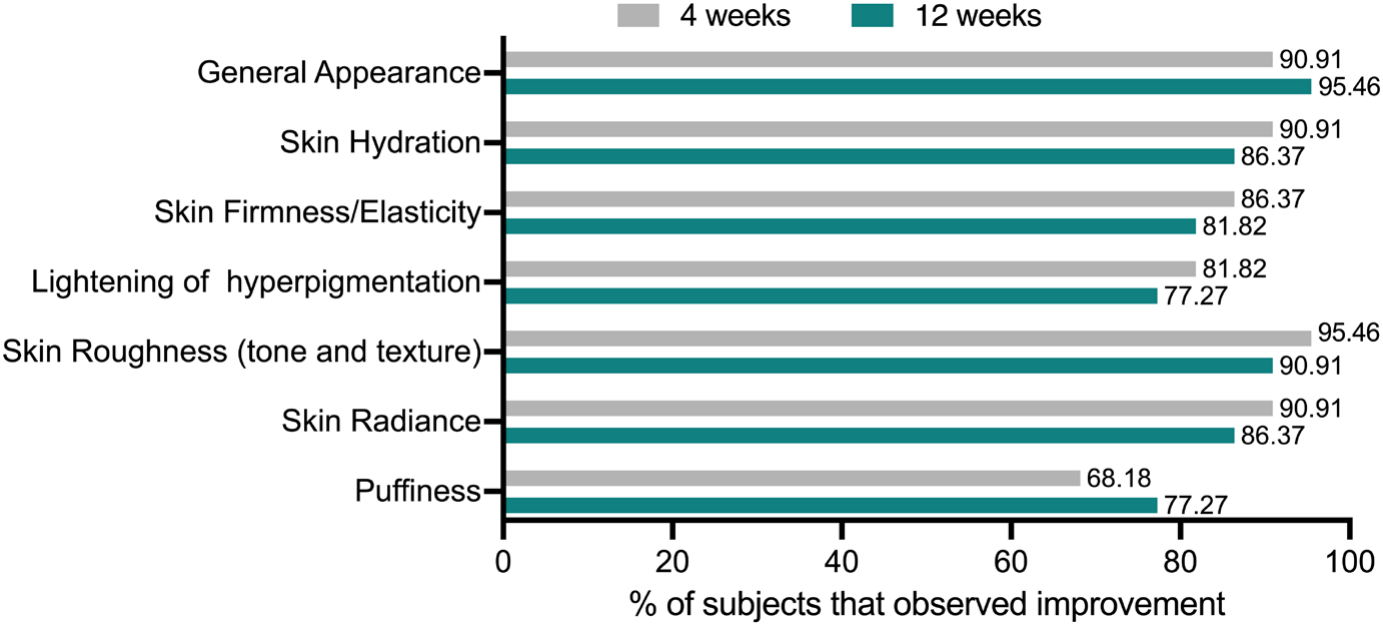
Subjective questionnaire results for week 4 and week 12. [Colour figure can be viewed at wileyonlinelibrary.com]

Each study participant completed a self‐assessment questionnaire to evaluate their overall satisfaction with OS‐01 EYE at weeks 4 and 12. At week 4, 90.91% of subjects reported feeling improvements in general appearance, increasing to 95.46% at week 12. Additionally, high percentages of participants noted increased skin hydration (90.91%), firmness and elasticity (86.37%), decreased hyperpigmentation (81.82%), smoother skin (95.46%), and improved skin radiance (90.91%) after 4 weeks of use. The improvement perception remained stable until 12 weeks. Improvements in puffiness around the eyes were also reported, with 68.18% of subjects showing improvements at week 4, increasing to 77.27% at week 12 (Figure 6).

## DISCUSSION

With a thinner epidermis and stratum corneum, the periorbital region signs of aging appear earlier than other facial areas, requiring specialized treatment [7, 9]. Previously, we have demonstrated the safety of the OS‐01 peptide both in vivo and ex vivo [12, 22] and its effects in preventing the accumulation of senescent cells and reducing DNA methylation age on human ex vivo models [12, 22]. This study demonstrates the ability of a new formulation of OS‐01 to provide a direct, effective, and safe mitigation of the phenotypes of aging in the periorbital area. Our results highlight the ability of OS‐01 EYE to effectively enhance multiple aspects of skin health and appearance over both short‐term (4 weeks) and long‐term (12 weeks) use, as assessed through instrumental measurements, expert grading, and self‐reported questionnaires.

The evaluation of skin barrier function, hydration, firmness, and elasticity using instrumental analysis allows objective skin analysis. Here, all bioinstrumental assessments demonstrated statistically significant improvements at both week 4 and week 12. TEWL values decreased and skin hydration increased significantly at both time points, as measured by evaporimeter and corneometer readings. TEWL, which refers to the amount of water that evaporates from the stratum corneum, is a common indicator of skin barrier function [23], regulating skin hydration levels and providing protection from external stressors [24]. The observed increase in skin hydration levels could result from both the improved skin barrier function and the direct effects of OS‐01 EYE. These results suggest that OS‐01 EYE enhances the skin's ability to retain moisture and protect against environmental stress over time. Previously published results on the OS‐01 peptide have also demonstrated its benefits in improving skin barrier integrity and hydration [13], and these effects may have been further enhanced by the addition of a higher concentration of OS‐01 peptide and other active ingredients in the formulation.

Skin firmness and elasticity also exhibited significant improvements, with enhancements observed as early as week 4 and further increases by week 12. Our previous research demonstrated that the OS‐01 peptide boosts collagen production, specifically increasing the gene expression of COL1A1 in both 3D skin equivalents and *ex vivo* skin, and contributes to increased epidermal thickness [12]. Additionally, in our prior double‐blinded, split‐face clinical study, improvements in wrinkle indentation and skin texture were found in areas treated with the OS‐01 peptide but not in areas treated with the vehicle control [13]. The clinical results from this study, combined with published findings, suggest that continued use of OS‐01 EYE leads to cumulative benefits in skin texture, likely due to the collagen‐boosting properties of OS‐01, with potential synergistic effects from other bioactives in the formulation, which are touted to increase collagen synthesis.

In addition to objective instrumental readings, results from blinded expert grading of clinical photographs and self‐reported participant questionnaires further validated skin changes during the study period. Blinded expert grading of the VISIA‐CR images revealed reductions in fine lines and wrinkles, improvement in skin smoothness, and decreased puffiness in around 50% of the participants. Self‐assessment questionnaires were administered to evaluate participants' experiences with OS‐01 EYE. Notably, 90.01% of the participants noted significant improvement in general skin appearance after 4 weeks, which increased to 95.46% by week 12. Although the percentage of participants reporting improvements remained high throughout the study, there was a slight decrease from week 4 to week 12 in some parameters, such as skin hydration, firmness/elasticity, lightening of hyperpigmentation, skin roughness, and radiance. We hypothesized that these minor declines in self‐reported data were due to the subjective nature of the questionnaires [25]. Participants may have scored lower for visible improvements at week 12 if they had already experienced significant enhancement by week 4.

Overall, this study's instrumental analyses, expert gradings, and self‐assessments provided compelling evidence that the OS‐01 EYE formula effectively targets signs of aging around the eyes. We verified through post‐hoc power analysis that our study was sufficiently powered for each outcome measured by instruments when comparing baseline to week 12. To further support the robustness and interpretability of our findings, we calculated Cohen's d for the four instrumental outcomes (skin hydration, firmness, elasticity, and TEWL) and for the three expert grading outcomes (fine lines and wrinkles, puffiness, dark circles). The effect sizes ranged from 0.64 to 1.52 (Table S4), corresponding to a medium to large effect across all outcomes, suggesting that our results are not only statistically significant but also practically significant. The combination of the high power and the substantial effect sizes supports the generalizability of our findings, at least within the specific population studied.

While our previous research demonstrated the benefits of the OS‐01 peptide in boosting collagen production, reducing DNA methylation age in vitro [12], and increasing hydration and skin barrier function in a previous clinical study with another formulation [13], the roles of other bioactive ingredients in the OS‐01 EYE formula should not be overlooked. The OS‐01 peptide concentration was increased by 25% compared to the formulation tested in the previous study, and ingredients such as *Fucus vesiculosus* extract, known for enhancing skin elasticity [16], hyaluronic acid for increased hydration [26], and *Moringa oleifera* Seed Extract for its antioxidative properties [27], may have contributed to the observed improvements and potentially enhanced the effects of the OS‐01 peptide. Further investigations with diverse and larger population size, as well as a no‐peptide control, are needed to isolate and confirm the specific effects of the OS‐01 peptide on periorbital aging. Nevertheless, overall, the OS‐01 EYE formulation is shown to be an effective treatment to significantly diminish the signs of periorbital skin aging.

## CONCLUSION

In the present study, the OS‐01 EYE formulation was shown to effectively reduce signs of aging in the delicate periorbital region, and enhance skin barrier function, hydration, firmness, and elasticity. There were no adverse events associated with the use of OS‐01 EYE. These findings suggest that OS‐01 EYE is a potent and safe formulation for addressing aging‐related skin concerns, with progressing improvements observed over 12 weeks of use compared to baseline measurements. This clinical study provides compelling evidence for using the OS‐01 peptide‐containing formulation as a pro‐longevity treatment for periorbital skin.

## CONFLICT OF INTEREST STATEMENT

AZ, MB, CRO, LEB, and JLC are named as inventors of a patent directed at this invention, which is solely owned by OneSkin, Inc. AZ, MB, CRO, and JLC are co‐founders of OneSkin Inc. CJ, JK, and SC are board‐certified advisors for OneSkin, Inc.

## ETHICAL APPROVAL

Before initiation, the study was approved by the Veritas Institutional Review Board (2022‐3082‐11740‐4), and informed consent was obtained from each volunteer prior to initiating the study. This clinical study was conducted in accordance with the International Conference of Harmonization Tripartite Guideline on Good Clinical Practice, applicable FDA regulations/guidelines set forth in 21 CFR Parts 11, and 50, and standard practices of ALS Beauty and Personal Care International Research Services, Inc.

## Supporting information


Figure S1.

Table S1.–S4.
