# Supplementary figures and images for: Clinical efficacy of OS‐01 peptide formulation in reducing the signs of periorbital skin aging
Source: Int J Cosmet Sci. 2025 Jan 9;47(3):455–65. doi: 10.1111/ics.13042 (PMC12127787; doi:10.1111/ics.13042)

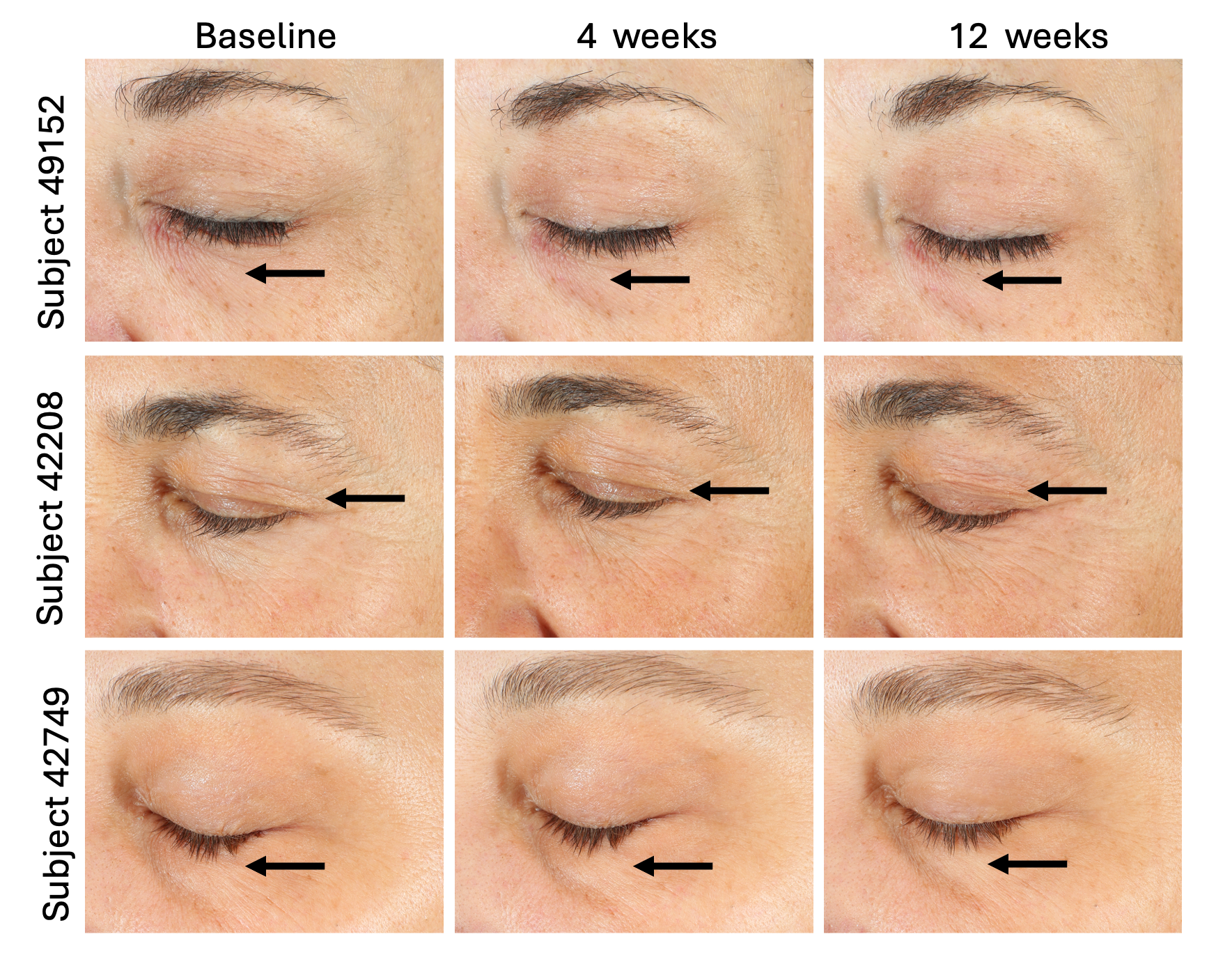

Supplement: Supplementary file 1 — Figure S1. [file ICS-47-455-s001.png]
