# Supplementary material for: Clinical efficacy of OS‐01 peptide formulation in reducing the signs of periorbital skin aging
Source: Int J Cosmet Sci. 2025 Jan 9;47(3):455–65. doi: 10.1111/ics.13042 (PMC12127787; doi:10.1111/ics.13042)
Supplement: Supplementary file 2 — Table S1.–S4. [file ICS-47-455-s002.docx]

**Figure S1.** Representative images of the left side of the eyes showing the skin-surface changes of the subjects

Supplementary Table 1: Inclusion and Exclusion Criteria

| **Inclusion Criterias** | - 1. Individuals who, at baseline, are free of any dermatological or systemic disorder, which would interfere with the results, at the discretion of the Investigator.   2. Individuals in good general health.   3. Individuals who complete a preliminary medical history.   4. Individuals who will read, understand and sign an informed consent document and photography release form.   5. Individuals who will be able to cooperate with the Investigator and research staff, have the test product applied according to the protocol and complete the full course of the study.   6. Individuals who are not concurrently participating in any other clinical study involving the same test site (face).   7. Individuals who have not participated in another facial clinical study in the past 14 days.   8. Individuals who will agree to discontinue use of personal care products (e.g. lotions, creams, serums, cleansers, sunscreen) for the washout period and duration of the study, with the exception of the products provided by ALS.   9. Individuals who agree to continue using approved cosmetics for the washout period and duration of the study so long as they do not contain anti-aging ingredients. ALS staff will review cosmetics for approval.   10. Individuals with visible fine line and/or wrinkles in the eye area (score 0.5-9 on a 10-point scale).   11. Individuals who agree to take a pregnancy test at the consent/screening and week 12 post-treatment visits. Individuals who agree not to sunbathe/tan and agree to avoid sun (UV) exposure as much as possible for the duration of the study. |
| --- | --- |
| **Exclusion Criterias** | 1. Individuals who have had a history of any acute or chronic disease that could interfere with or increase the risk on study participation. 2. Individuals with an active (flaring) disease or chronic skin allergies (atopic dermatitis, eczema, and psoriasis), or had recently treated skin cancer (within the last 12 months). 3. Individuals with a history of immunosuppression/immune deficiency disorders or currently using immunosuppressive medications (e.g., azathioprine, belimumab, cyclophosphamide, Enbrel, Imuran, Humira, mycophenolate mofetil, methotrexate, prednisone, Remicade, Stelara.) and/or radiation as determined by study documentation. 4. Individuals with damaged skin at or in close proximity to test sites (e.g., sunburn, tattoos, scars, or other disfigurations). 5. Individuals who have any history, which, in the Investigator's opinion, indicates the potential for harm to the subject or could place the validity of the study in jeopardy. 6. Individuals who indicate that they are pregnant, planning a pregnancy or nursing. Individuals must be employing a medically effective form of birth control for at least 3 months prior to the study and must agree to not change their form of birth control during study conduction. Medically effective contraception includes hormonal methods, such as the contraceptive pill or implant, or an intrauterine device (IUD) in use at least 30 days before study product administration, or barrier methods such as diaphragm plus spermicide or condom plus spermicide, in use at least 14 days before study product administration. 7. Individuals who have been medically diagnosed with Type I Diabetes. 8. Individuals who have had any medical procedure, such as laser resurfacing, or plastic surgery to the test sites within the last 2 years (including Botox, collagen injections, Restylyn, or other fillers). 9. Individuals who are currently using or during the last 3 months have used, Retin A, or other Rx/OTC Retinyl A, or other skin lightening / astringent derived products or alpha hydroxyl acid treatments for photo-aging and fine lines/wrinkles. 10. Individuals who have started hormone replacement therapy in the past 3 months. 11. Individuals who have a known history of hypersensitivity to any cosmetics, personal care products, and/or fragrances. 12. Individuals who are employees of ALS. |

Supplementary Table 2: Ingredient list of experimental product

| **Formulation** | **Ingredient list** |
| --- | --- |
| OS-01 EYE formulation | Water, Glycerin, Sorbitan Olivate, Squalane, Cetearyl Olivate, Butyrospermum Parkii (Shea) Butter, Simmondsia Chinensis (Jojoba) Seed Oil, Cystoseira Humilis Extract, d-Panthenol, Bentonite, Cetyl Palmitate, Sodium Hyaluronate Crosspolymer, Caprylhydroxamic Acid, Sorbitan Palmitate, Cellulose, Coffea Arabica (Coffee) Seedcake Extract, Allantoin, Fucus Vesiculosus Extract, Gluconolactone, Niacinamide, sh-Polypeptide-121, Sisymbrium Irio Seed Oil, Tocopheryl Acetate, Sorbic Acid, Caprylic/Capric Triglyceride, Ubiquinone, Lecithin, Decapeptide-52*, Sodium Chloride, Maltodextrin, Acetyl Hexapeptide-8, Moringa Oleifera Seed Extract, Glyceryl Caprylate, Caprylyl Glycol, Tetrasodium Glutamate Diacetate, Alcohol, Sodium Benzoate, Phenoxyethanol, Xanthan Gum. *OS-01 Peptide. |

Supplementary Table 3: Expert Grading Parameters

| **Parameter** | **Scale** |
| --- | --- |
| Eye Area Lines / Wrinkles | 7-9 = Severe appearance of fine lines/wrinkles  4-6 = Moderate appearance of fine lines/wrinkles  1-3 = Mild appearance of fine lines/wrinkles  0 = No fine lines/wrinkles |
| Eye Area Puffiness | 7-9 = Severe appearance of puffiness  4-6 = Moderate appearance of puffiness  1-3 = Mild appearance of puffiness  0 = No appearance of puffiness |
| Eye Area Dark Circles | 7-9 = Severe appearance of dark circles  4-6 = Moderate appearance of dark circles  1-3 = Mild appearance of dark circles  0 = No appearance of dark circles |

Supplementary Table 4: Summary results from post-hoc power analysis

|  | Paired t-test | Paired t-test with 2000 Monte Carlo simulations | Cohen's d |
| --- | --- | --- | --- |
| Objective measures |  |  |  |
| Hydration | 1.000 | 1.000 | 1.52 |
| Firmness | 0.811 | 0.815 | 0.64 |
| Elasticity | 0.998 | 0.999 | 1.10 |
| TEWL (barrier function) | 0.881 | 0.875 | 0.70 |
| Expert grading |  |  |  |
| Fine lines and wrinkles | 0.996 | 0.998 | 1.03 |
| Puffiness | 0.989 | 0.991 | 0.95 |
| Dark circles | 0.975 | 0.980 | 0.88 |
